# Fusion of Magnetic Resonance Elastography Images With Computed Tomography and Magnetic Resonance Imaging Using the Human Visual System

**DOI:** 10.7759/cureus.45109

**Published:** 2023-09-12

**Authors:** Fayez M Alharbi, Faten R Besbes, Basim S Almutairi, Abdullah T Alotaibi, Faiq F Fatani, Hatem R Besbes

**Affiliations:** 1 Physics, King Abdulaziz University, Jeddah, SAU; 2 Engineering, University of Sfax, Sfax, TUN; 3 Medical Imaging, King Saud Medical City, Riyadh, SAU

**Keywords:** discrete wavelet transform (dwt), contrast sensitivity function (csf), magnetic resonance elastography (mre), multimodality medical image fusion (mmif), human visual system (hvs)

## Abstract

Magnetic resonance elastography (MRE) is used to assess the stiffness of the liver to rule out cirrhosis or fibrosis. The image, nevertheless, is regarded as shear-wave imaging and does not depict any anatomical features. Multimodality medical image fusion (MMIF), such as the fusion of MRE with computed tomography (CT) scan or magnetic resonance imaging (MRI), can help doctors optimize the advantages of each imaging technique. As a result, perceptions serve as valid and valuable assessment criteria. The contrast sensitivity function (CSF), which describes the rates of visual contrast sensitivity through the changing of spatial frequencies, is used mathematically to characterize the human visual system (HVS). As a result, we suggest novel methods for fusing images that use discrete wavelets transform (DWT) based on HVS and CSF models. Images from MRI or CT scan were combined with MRE images, and the outcomes were assessed both subjectively and objectively. Visual inspection of merging images was done throughout the qualitative analysis. The CT-MRE fused images in all four datasets were shown to be superior at maintaining bones and spatial resolution, despite the MRI-MRE being better at exhibiting soft tissues and contrast resolution. It is clear from all four datasets that the liver soft tissue in MRI and CT images mixed successfully with the red-colored stiffness distribution seen in MRE images. The proposed approach outperformed DWT, which produced visual artifacts such as signal loss. Quantitative evaluation using mean, standard deviation, and entropy showed that the generated images from the proposed technique performed better than the source images and DWT. Additionally, peak signal-to-noise ratio, mean square error, correlation coefficient, and structural similarity index measure were employed to compare the two fusion approaches, namely, MRI-MRE and CT-MRE. The comparison did not show the superiority of one approach over the other. In conclusion, both subjective and objective evaluation approaches revealed that the combined images contained more information and characteristics. Hence, the proposed method might be a useful procedure to diagnose and localize the stiffness regions on the liver soft tissue by fusion of MRE with MRI or CT.

## Introduction

Medical imaging is becoming increasingly crucial in a variety of clinical applications, such as diagnosis, treatment, and surgery. Because of the diversity of imaging technologies, multimodal diagnostic images with several modalities may contribute to obtaining more information about different body parts [1-3]. For instance, magnetic resonance elastography (MRE) is a relatively new method for imaging and measuring the mechanical characteristics of tissues. This magnetic resonance imaging (MRI) sequence called phase contrast (PC) calculates the shear wave motion. Furthermore, it is a non-invasive method, and unlike other modalities, it is also used to measure hepatic stiffness and assess the early stages of liver fibrosis and cirrhosis. However, MRE imaging is thought of as a shear-wave image and does not immediately depict anatomical details or the precise location of the lesions as it represents the mechanical properties of the organ or tissue and does not show the anatomy or soft tissues [4-6]. MRI provides high-resolution anatomical information about soft tissues, whereas computed tomography (CT) scans, for example, can precisely detect dense structures such as bones [7-9]. The advantages of each modality may be observed in fused images such as MRI-MRE and CT-MRE. Nevertheless, the multimodality medical image fusion (MMIF) method, which aims to generate a composite image to integrate complementary information, efficiently collects sufficient information for an accurate diagnosis [10].

Image processing techniques have been used to enhance the visual quality of images for the past few decades. Digital image fusion is considered one of the novel image processing techniques to merge two images from various modalities into one image [11,12]. More information is contained in the resulting fused image than in any original image. The best results are seen in fusion when using the human visual system (HVS) model for image processing. Among the characteristics of HVS are sensitivity, brightness adaption level, and texture activity. The contrast sensitivity function (CSF), which depends on contrast and spatial frequency, describes the pattern sensitivity of the HVS. The HVS weights for each detail band and an approximation band are computed using the CSF. Quantized HVS weights can gather the most important information. Because the HVS interprets information in a multi-resolution manner, multi-scale transforms (MST), such as the discrete wavelets transform (DWT), can typically produce perceptually pleasing results as it is similar to HVS’s multiple channel models. It also helps locate crucial perceptual information, decreasing spatial distortions and spectral degradations formed in the merged image, as well as resulting in less computation time [13-23].

In general, wavelet transform (WT) depends on wavelets, as opposed to Fourier transform, whose fundamental functions are sinusoids. A finite-length waveform known as the mother wavelet is represented by wavelets, which are mathematical functions. A signal (image) is broken down into several frequency components at various resolution scales using the wavelet transform (also known as multi-resolution). This makes it possible to simultaneously disclose an image’s spatial and frequency properties.

In DWT, an image can be generated by surpassing it via a filter bank investigation and then destroying it. This filter bank investigation is frequently used in image processing and comprises a low-pass filter (approximation subband) and a high-pass filter (detail subband) at every decomposition stage. When an image undergoes these filters, they separate into two processing groups. The low-pass filter removes the coarse data from the image while maintaining a relationship with an averaging capacity. The high-pass filter extracts the image’s coarse details by maintaining correspondence to a varying capability. Following that, the yield of the separating capabilities is divided by two. DWT employs a variety of filters for signal processing, including the Daubechies, Coiflet, Haar, reverse biorthogonal, biorthogonal, Fejer-Korovkin wavelets, symlets, and discrete approximation of Meyer wavelet. This study used the Haar wavelet, which is known as the earliest and most basic wavelet family. It resembles a step function and is discontinuous [24-30].

The vast majority of previous studies used DWT + HVS to study watermarking applications, image compression, and, recently, image fusion. Regarding image fusion studies, most previous studies selected brain exams such as CT and MRI for fusion research. It is logical as the brain is easier compared to other parts such as the abdomen in terms of pre-processing such as image registration and noise reduction. Moreover, the availability of considerable previous research in brain fusion is very helpful for comparison. However, no previous studies on fusion liver MRI or CT with MRE using DWT + HVS are available to compare the results of this study with the. For this investigation, the liver was chosen as part of interest and the chosen modalities were CT and MRI. The purpose of this study is to fuse the MRE image with MRI or CT image using DWT based on HVS to model CSF. It also used four groups of images involving studies with liver abnormalities such as tumors, cirrhosis, and other conditions.

## Technical report

MMIF involves the following three steps: (1) pre-processing, (2) processing, and (3) post-processing. The pre-processing step includes selecting four datasets of MRI, CT, and MRE images for the liver. The selected imaging machines for this study were an MRI machine (GE Optima MR 450w, Boston, MA, USA) and a CT machine (GE Discovery CT750 HD, Boston, MA, USA). It also comprises image registration. The MRI and CT images of the liver are utilized as the source images, whereas the MRE image is considered the target. It was done by MATLAB programming (MathWorks, Natick, MA, USA), and the Feature-Based Image Registration algorithm was the method of choice. In detail, first, it includes feature detection between the source and target images such as corners and boundaries. Second, it matches the corresponding regions between the two images. Subsequently, it applies the affine transformation rule, and, lastly, it creates the registered image.

**Figure 1:**
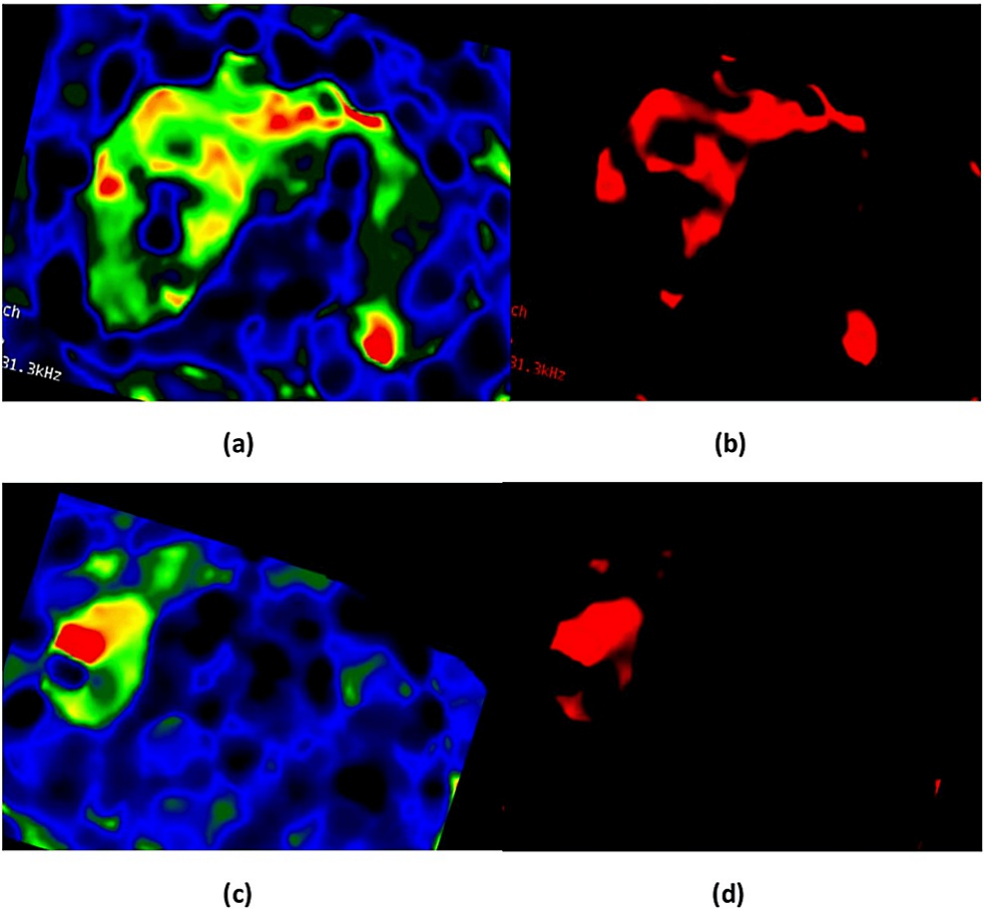
MRE images before (a and c) and after (b and d) the extraction of abnormal regions. MRE = magnetic resonance elastography

A color wave image contains several colors (burble, blue, green, yellow, orange, and red). These colors represent the elasticity levels, either in normal or abnormal ranges. We were only interested in the stiffness areas or abnormal ranges in the MRE image. Hence, we ran MATLAB codes to only extract the abnormal regions (yellow, orange, and red) from the RGB image and changed the pixel values for the other colors to zero or black (see Figure 1). As a result, the normal regions appeared black, while the abnormal regions appeared red in color. The advantage of this is being able to extract tiny and early stages of stiffness, fibrosis, and cirrhosis. In a normal study of MRE, the extracted image will be completely black, which may be a useful way to clearly see the development after treatment.

Second, the processing step is the fusion of the registered images (MRI or CT with MRE). It uses DWT based on HVS-CSF and is processed by MATLAB programming (R2018a). In detail, each sub-block in the fusion process undergoes a transformation to a sub-band up to the fifth level of DWT decomposition. Each decomposition comprises four sub-bands (low-resolution sub-images) in the transformed space. One is considered approximation (LL) and three are known as details (LH, HL, and HH) (L: low-pass filtered, H: high-pass filtered). The CSF curve is similarly broken down to the fifth level, and the weights of each sub-band are determined. Each and every DWT sub-band gets multiplied by the corresponding CSF weights. The sum of all sub-band weights yields the overall sub-band weight. The weights of the sub-bands are compared, and the appropriate sub-bands are chosen from the source images. Similarly, all sub-bands are chosen. The inverse discrete wavelet transform is then used to turn the combined image back into a spatial domain. In conclusion, All DWT domain sub-band HVS weights are computed. Each band’s outcome is computed using the HVS weights of the following band. For image fusion, the highest bands from the results are chosen. The resultant fused image is an MRI or CT of the liver with MRE (with stiffness areas)). It will be a grayscale image showing the soft tissue of the liver with abnormal stiffness areas such as fibrosis or cirrhosis. For fusion, we were only interested in abnormal regions in the MRE image (not-anatomic) to localize them on liver soft tissue in MRI or CT images (anatomic); hence, in the fused images, we would be able to see the normal liver soft tissue as it appeared in MRI or CT images with stiffness regions from the MRE image.

**Figure 2:**
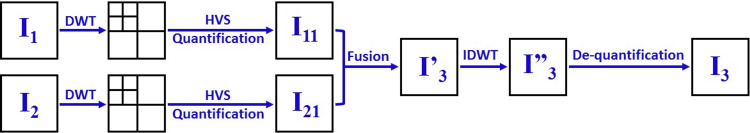
Block diagram of the proposed method to fuse two images using DWT + HVS. DWT = discrete wavelet transform; IDWT = inverse discrete wavelet transform; HVS = human visual system

**Figure 3:**
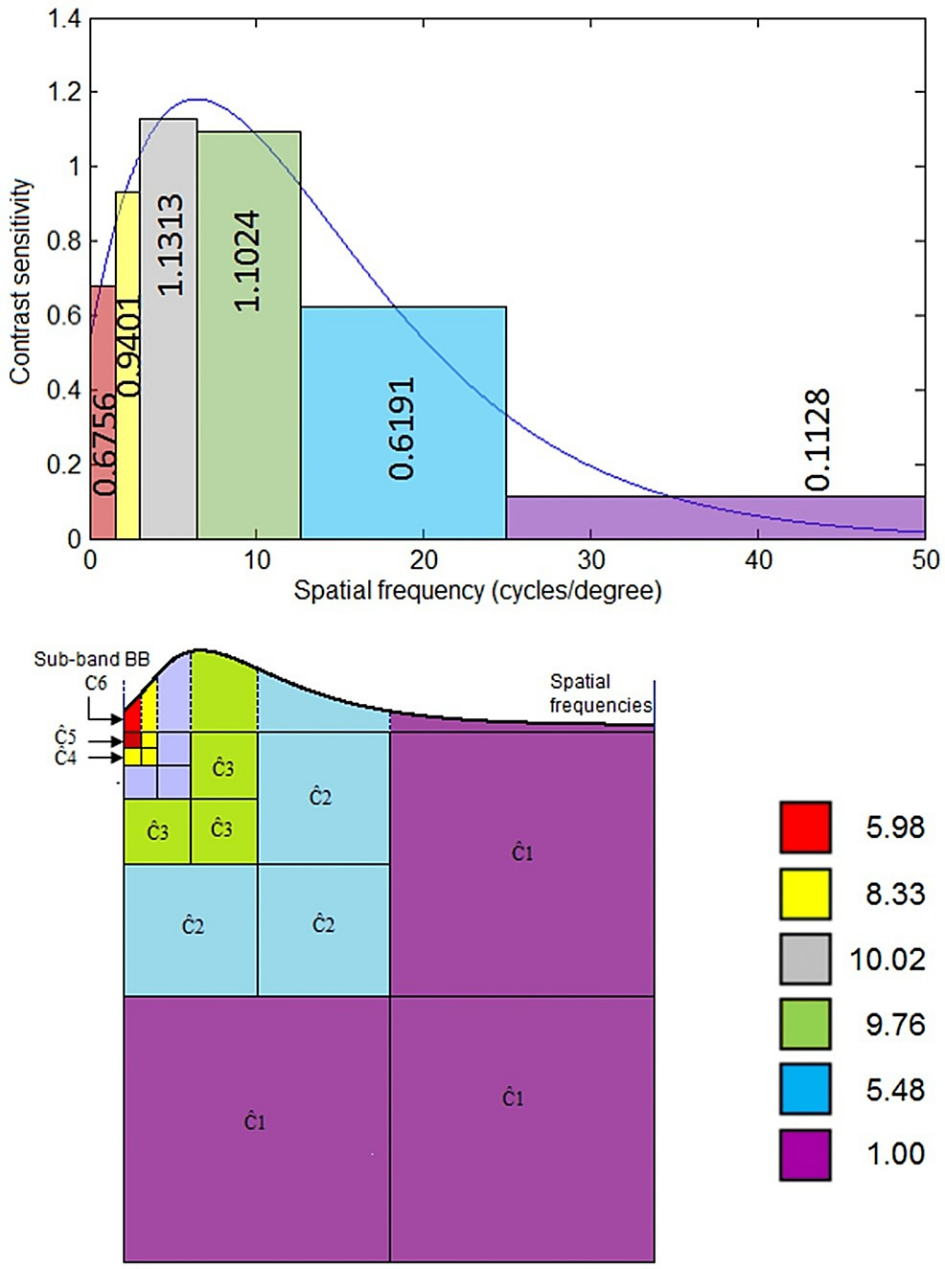
Calculation of quantification weights by CSF decomposition. The figure shows the adopted wavelet coefficient for fusion using HVS and CSF. CSF reaches its highest value between sub-bands C3 and C4. HVS = human visual system; CSF = contrast sensitivity function; C1-6 = coefficients (hcsf  and wavelet sub-band)

In particular, the suggested method in Figure 2 illustrates the fusion process of two images. Figure 3 shows optimizing quantification using the CSF function adjusted to the HVS. For each wavelet sub-band, the quantified factors show the HVS’s frequency sub-band average sensitivity. The frequency bands are formed using wavelet decomposition as an analog. The rate of the spatial frequency CSF values for each sub-band determines the value of the $h_{csf}$ coefficients. After normalizing the coefficients, the lowest rate of  $h_{csf}$ is set to one. Additionally, the coefficients $h_{csf}$ are computed using equation (1) as follows:

$h_{csf}(\lambda )=\int_{i}^{j}csf(f)$

Where the spatial frequency $f$ is expressed in cycles for every degree, and $i, j$ are the symbols of the boundaries of every subband $CSF(f)$ of the CSF.

We assumed another CSF mask as the following equation (2):

$w_{csf}(\lambda )=\frac{1}{h_{csf(\lambda )}}$

The sensitivity rate of the $h_{csf(\lambda )}$ on the frequency band associated with every wavelet transform sub-band is inversely proportional to the modeling coefficients of $w_{csf(\lambda )}$. For a fixed interval, the spatial frequencies of the $h_{csf(\lambda )}$ increase in prominence while the coefficient of $w_{csf(\lambda )}$ decreases.

Lastly, the post-processing step includes image evaluation. It is divided into subjective and objective evaluation.

Subjective evaluation

Subjective evaluation was performed by two experienced radiologists. The evaluation aimed to compare their similarities and dissimilarities.

Objective evaluation

The fused images were compared with source images regarding their similarities and dissimilarities. As we do not have reference images, statistical parameters such as standard deviation (σ), mean (μ), and entropy (En) were selected as metrics for assessments. In addition, the fusion modalities MRI-MRE and CT-MRE were evaluated using other image quality metrics such as peak signal-to-noise ratio (PSNR), mean square error (MSE), correlation coefficient (CC), and structural similarity index measure (SSIM).

## Discussion

**Figure 4:**
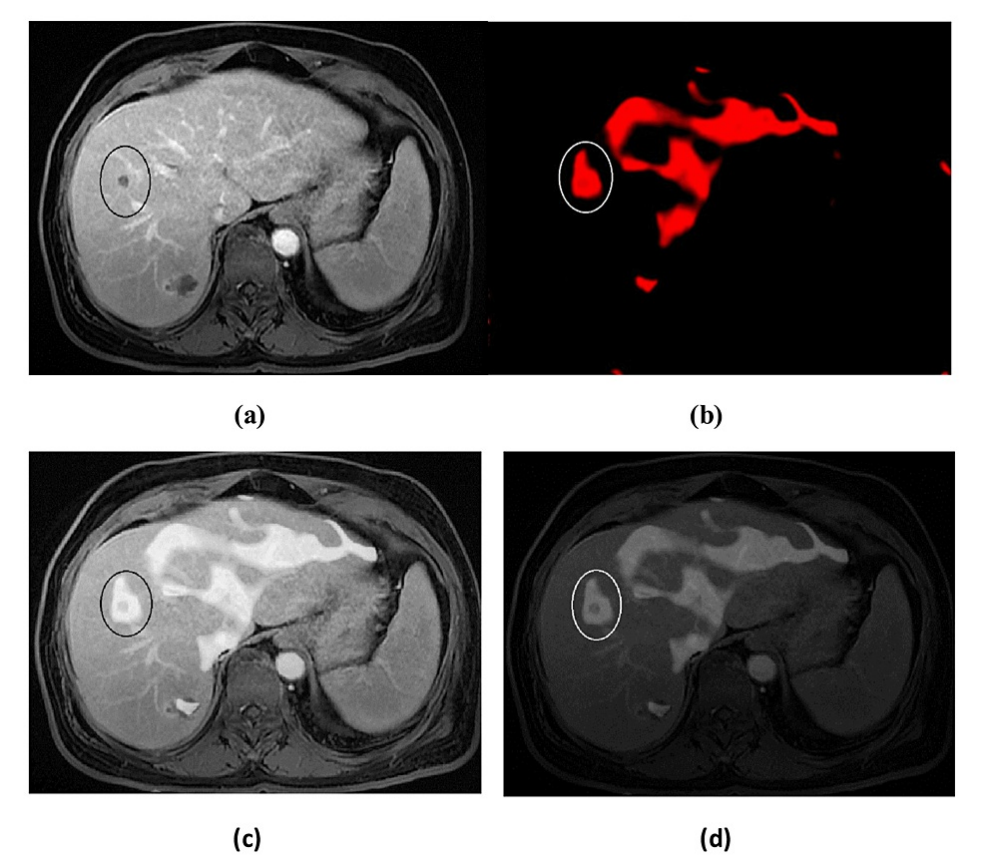
MRI-MRE fusion. (a) Liver MRI. (b) Liver MRE. (c) Bright fused image. (d) Dark fused image. MRI = magnetic resonance imaging; MRE = magnetic resonance elastography

**Figure 5:**
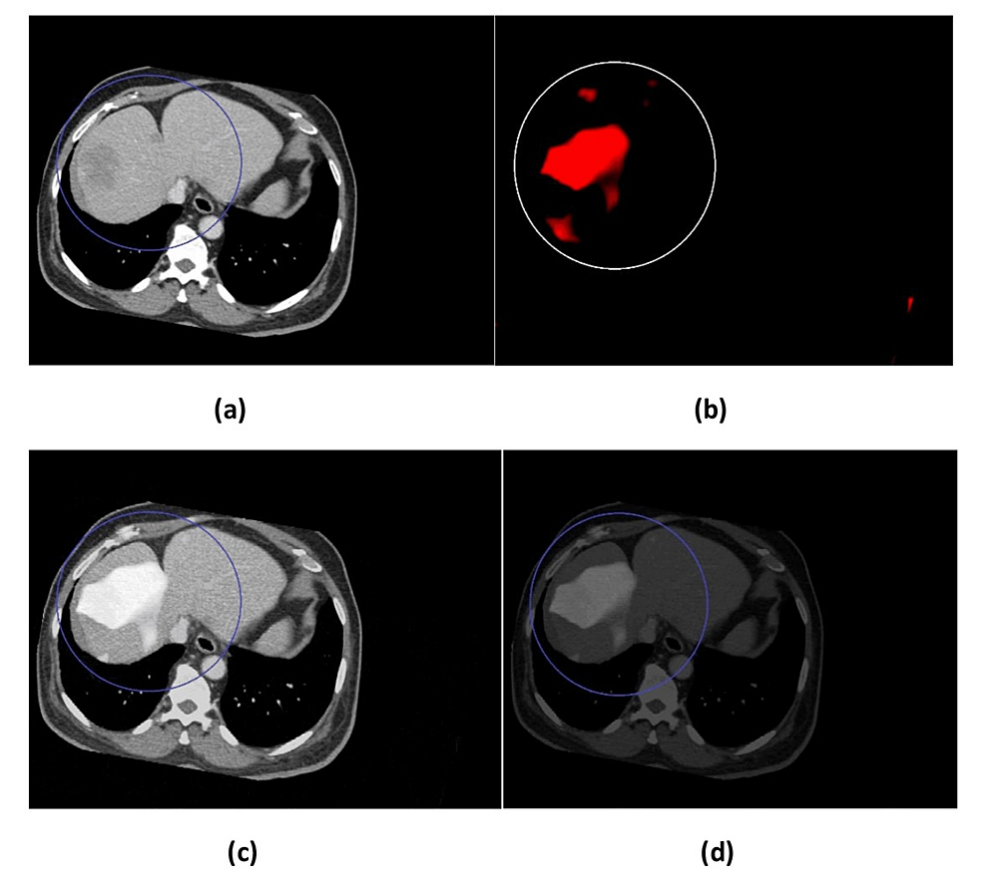
CT-MRE fusion. (a) Liver CT scan. (b) Liver MRE. (c) Bright fused image. (d) Dark fused image. CT = computed tomography; MRE = magnetic resonance elastography

**Figure 6:**
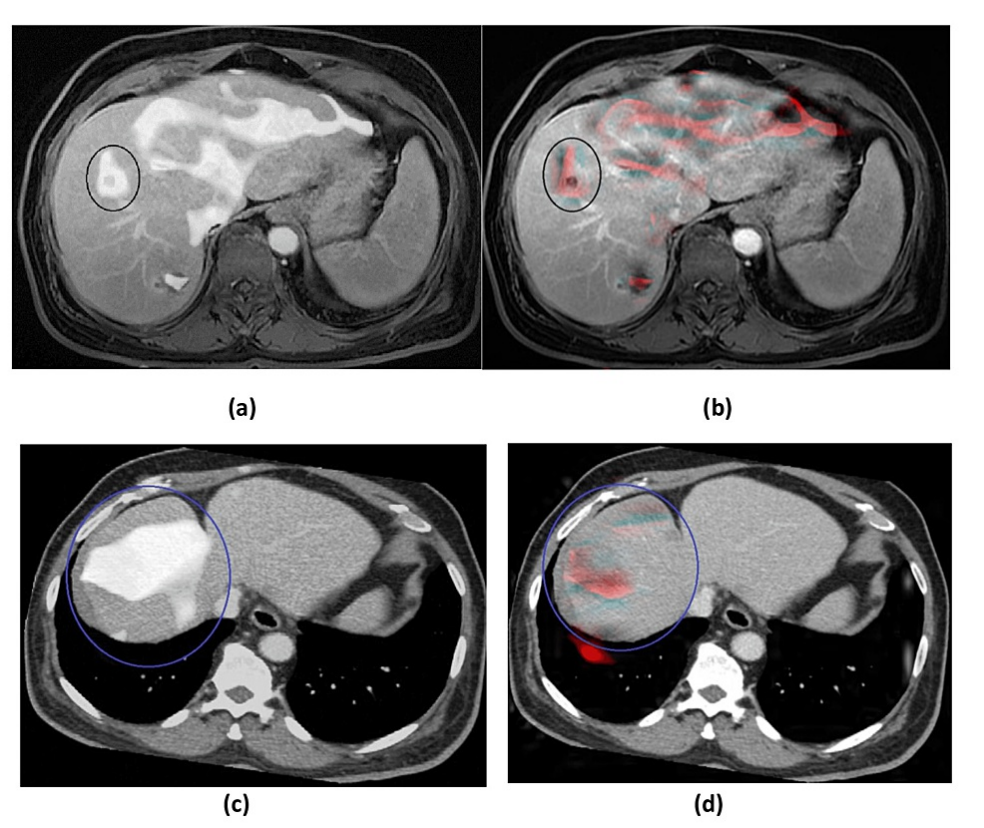
Comparison between the proposed method and DWT fusion. (a) Proposed method (MRI-MRE fusion), (b) DWT (MRI-MRE fusion), (c) Proposed method (CT-MRE fusion), (d) DWT (CT-MRE fusion). CT = computed tomography; MRI = magnetic resonance imaging; MRE = magnetic resonance elastography; DWT = discrete wavelet transform

The fused images in Figure 4 and Figure 5 and the comparison between the proposed method and DWT in Figure 6 were evaluated by two different methods, i.e., subjective and objective evaluation. Four groups of images with liver abnormalities, such as tumors and cirrhosis, were taken for this study.

Subjective evaluation

Subjective evaluation was done by two experienced radiologists. The evaluation of fused images (Figures 4, 5) showed that the resultant fused images had more information as they involved the structural MRI or CT and MRE images together, which gave better results. In detail, the fused image contained the stiffness areas in the elastogram image as well as anatomic MRI or CT with contrast, which provided a comprehensive and homogenous fused grayscale image. Regarding the comparison between the proposed method and conventional DWT in Figure 6, it can be clearly seen that the proposed method was better and the final fused image had more information and no loss of signal. However, the subjective procedures rely on psycho-visual testing, cannot be implemented automatically, and are regarded as complicated. Additionally, the difficulty in fusing images due to interobserver variability necessitates the employment of a quantitative parameter evaluation to assess the effectiveness of fused images.

Objective evaluation

As shown in Table 1, these statistical values were higher for combined images compared to the source images in all four groups. The higher values of the three parameters (μ, σ, and En) revealed that the qualities of fused images were better compared to the source images (CT, MRI, and MRE). Further, the vast majority of outcomes showed that the qualities of fused images of the proposed method were higher in all four datasets than in those of the DWT method. The MRI-MRE and CT-MRE merged images are evaluated in Table 2 using other image quality metrics such as PSNR, MSE, CC, and SSIM. It showed that the results varied between the two fusion approaches.

**Table 1:** Statistical parameters for objectively evaluating and comparing the proposed method with DWT and comparing the fused images (MRI + MRE and CT+MRE) with the source images (MRI, CT, and MRE). μ = mean; σ = standard deviation; En = entropy; CT = computed tomography; MRI = magnetic resonance imaging; MRE = magnetic resonance elstography; DWT = discrete wavelet transform

EN	σ	μ	Image	Group
5.0688	61.8567	80.9972	CT	Set 1
5.62594	64.5863	74.9730	MRI	
1.0395	18.7961	5.2710	MRE	
6.3767	75.4642	75.4217	Fused (CT + MRE) - DWT	
6.4132	63.9975	83.8610	Fused (CT + MRE) - DWT (Proposed method)	
5.7252	61.0440	76.9290	Fused (MRI + MRE) - DWT	
5.9624	65.2931	89.8551	Fused (MRI + MRE) - DWT (Proposed method)	
4.8114	74.9660	75.7672	CT	Set 2
4.3162	58.2635	73.4217	MRI	
0.7066	13.2940	2.4062	MRE	
5.3767	75.4642	75.4217	Fused (CT + MRE) - DWT	
4.8318	77.2894	75.8735	Fused (CT + MRE) - DWT (Proposed method)	
4.3208	63.4112	81.4723	Fused (MRI + MRE) - DWT	
4.33325	66.8751	87.8541	Fused (MRI + MRE) - DWT (Proposed method)	
4.3507	70.5764	71.0212	CT	Set 3
5.2084	66.2635	75.6000	MRI	
1.2969	21.2984	7.4351	MRE	
4.4634	70.5421	77.1210	Fused (CT + MRE) - DWT	
4.3832	75.7642	80.3740	Fused (CT + MRE) - DWT (Proposed method)	
5.3527	70.0140	83.4572	Fused (MRI + MRE) - DWT	
5.2178	76.3492	88.6542	Fused (MRI + MRE) - DWT (Proposed method)	
4.7562	76.3404	82.356	CT	Set 4
4.4876	70.2635	73.4400	MRI	
1.9526	33.2184	11.0451	MRE	
5.1597	80.4764	85.6550	Fused (CT + MRE) - DWT	
5.1907	83.5011	87.7921	Fused (CT + MRE) - DWT (Proposed method)	
4.6273	65.1750	75.6021	Fused (MRI + MRE) - DWT	
4.5014	69.4513	77.6518	Fused (MRI + MRE) - DWT (Proposed method)	

**Table 2:** Comparison between MRI-MRE and CT-MRE fusion methods. PSNR = peak signal-to-noise ratio; MSE = mean square error; CC = correlation coefficient; SSIM = structural similarity index measure; MRE = magnetic resonance elastography; MRI = magnetic resonance imaging; CT = computed tomography

SSIM	CC	MSE	PSNR	Parameter	
				Fusion method	Group
0.9751	0.8682	37.83	38.66	MRI-MRE	Set 1
0.9727	0.8570	37.32	36.81	CT-MRE	
0.9762	0.8581	39.55	39.09	MRI-MRE	Set 2
0.9737	0.8543	37.41	37.00	CT-MRE	
0.9639	0.9285	47.33	47.26	MRI-MRE	Set 3
0.9689	0.9110	47.11	47.72	CT-MRE	
0.9754	0.9207	36.69	37.39	MRI-MRE	Set 4
0.9663	0.8703	37.04	37.71	CT-MRE	

The resultant images (see Figures 4-6) were evaluated qualitatively and quantitatively. In qualitative analysis, it was done by human visual inspection of merged images. The CT-MRE fused images in all four datasets were better in preserving bones and spatial resolution, whereas the MRI-MRE images were superior in displaying soft tissues and contrast resolution. In general, CT and MRI of the liver with contrast are the best methods of choice to view the enhancement of tumors and lesions. On the other hand, MRE represents the mechanical properties and shows the stiffness distribution. Particularly, this fusion technique provided all this information in one homogenous grayscale image. In all four datasets, it is clearly seen that the texture of stiffness distribution present in MRE images, represented by red color (as shown in Figure 4b and Figure 5b), was merged with liver soft tissue in the MRI image in Figure 4a and the CT image in Figure 5a. The removal of normal areas (non-regions of interest (ROIs)) and preservation of abnormal areas (ROIs) in MRE images, as shown in Figure 1, was very helpful in obtaining the fused images for the normal liver soft tissue as it appeared in MRI and CT images with only abnormal regions (stiffness distribution) as it appeared in MRE images. Thus, it is robustly successful in getting a homogenous grayscale image, as shown in Figure 4c and Figure 5c, with more information than the source images (CT, MRI, and MRE). Figure 6 shows the comparison between the proposed method and DWT for CT-MRE and MRI-MRE fused images. It was observed that the fused images of the proposed method in Figure 6a and Figure 6c were better than those of DWT in Figure 6b and Figure 6d. In the proposed method, the stiffness areas in MRE images did not change after the fusion process. Their textures and shapes were the same before and after. However, we changed the stiffness area colors to a grayscale with the goal of being consistent with MRI or CT grayscale images. As a result, this did not affect the final result as the MRE images had only one color (red) representing the stiffness areas and they did not change the textures and shapes of stiffness regions. On the other hand, in DWT, the red color of MRE images was the same before and after the fusion. However, the textures, appearances, and shapes of the stiffness areas of MRE changed after the fusion process. Furthermore, there were visual artifacts such as darkness or loss of signal and edge inconsistency in some regions in all DWT images after fusion.

In quantitative analysis (see Table 1), the resultant images in all four datasets appeared to have more information in all three parameters, i.e., mean, standard deviation, and entropy. Datasets 1 to 4 comprise source images such as CT, MRI, and MRE. They also contain the fused images of the proposed method and DWT for both fusion approaches, CT-MRE and MRI-MRE. In dataset 1, the three parameters were more in both fusion approaches of the proposed method compared to source images and DWT, with the exception of the standard deviation of CT-MRE images, which was higher in DWT than the proposed methods. There are slight differences between the CT and MRI images and the fused images of the proposed method. However, it is clearly seen that a significant variation was present between MRE and the fused images. As the mean was much higher in the proposed fused method compared to the MRE images, the brightness of the resultant image was much higher. Standard deviation, on the other hand, had a significant increase in the merged images compared to the MRE images, which indicated that the contrast was extremely high. Furthermore, the entropy of fused images provided more information as it was much more than those of MRE images. It was also higher in the fused DWT images than those of the source images, and it is reasonable as the fused images had more information. Similarly, databases 2, 3, and 4 showed that the statistical results of fused images of the proposed methods were higher than those of source images and the DWT method. In all databases, entropy was higher in the DWT compared to the CT, MRI, and MRE images. Meanwhile, the mean and standard deviation had a certain fluctuation in those images. Furthermore, Table 2 demonstrates multimodal fusion methods used in this study, such as MRI-MRE and CT-MRE, which were compared by other image quality metrics such as PSNR, MSE, CC, and SSIM. In databases 1 and 2, these four parameters were higher in MRI-MRE fused images than those in CT-MRE images. In dataset 3, PSNR and SSIM were higher in CT-MRE than those in MRI-MRE merged images. MSE and CC, on the other hand, were slightly higher in MRI-MRE images. Lastly, in database 4, PSNR and MSE were higher in CT-MRE, while CC and SSIM were higher in MRI-MRE images. As a result, the comparison shown in Table 2 could not determine which fusion strategy produced the best outcomes. In conclusion, both subjective and objective evaluation methods revealed that the fused images of the proposed method displayed more information and detail than the source images, and they were very helpful in localizing the stiffness regions on the liver soft tissue in a composite image. Moreover, the results of the proposed method were better when compared to DWT.

## Conclusions

This study proposed a novel method for fusing medical images considering the HVS properties. It includes CSF function modulation in the process of multimodal fusion with DWT. MRI and CT liver images were fused with MRE images. The fused images were evaluated by subjective and objective evaluation. In qualitative analysis, merged images were visually inspected. It was concluded that although MRI-MRE was better at demonstrating soft tissues and contrast resolution, the CT-MRE fused images in all four datasets were better at maintaining bones and spatial resolution. In all four datasets, the red-colored stiffness distribution texture observed in MRE images blended efficiently with the liver soft tissue in MRI and CT images. The suggested method was also superior to DWT, which resulted in visual artifacts including signal loss. The suggested method’s generated images outperformed the source images and DWT in quantitative analyses, including mean, standard deviation, and entropy.

Furthermore, PSNR, MSE, CC, and SSIM were used to compare the two MRI-MRE and CT-MRE fusion techniques. These four metrics were higher in MRI-MRE fused images in datasets 1 and 2. In dataset 3, MRI-MRE had higher MSE and CC, while CT-MRE had higher PSNR and SSIM. In dataset 4, MRI-MRE images had higher CC and SSIM, while CT-MRE images had higher PSNR and MSE. This meant that the comparison did not show which fusion approach was better as the results varied between the two approaches. In conclusion, the combined images had more information and details, as shown by both subjective and objective evaluation techniques, and the proposed method was better when compared to DWT.

The proposed method might be very helpful to doctors for improved visualization, precise interpretation, and accurate localization of the stiffness areas in the liver soft tissue. However, a prospective study should be conducted as this study depended on the previous examinations from databases, which were done on different unknown conditions. It is logical to perform all imaging procedures for each patient within a short time and take all measurements into consideration to increase accuracy. Further research must be done in the future. Artificial intelligence programming such as deep learning and neural networks may be used for further improvement. Mover, the larger datasets can be collected in a longer sufficient time.
